# The roles of TGF-β and VEGF pathways in the suppression of antitumor immunity in melanoma and other solid tumors

**DOI:** 10.1016/j.pharmthera.2022.108211

**Published:** 2022-05-14

**Authors:** Melissa T. Bu, Pallavi Chandrasekhar, Lizhong Ding, Willy Hugo

**Affiliations:** a Department of Medicine/Dermatology, University of California Los Angeles, Los Angeles, CA 90095, USA; b Department of Molecular, Cell, and Developmental Biology, University of California Los Angeles, Los Angeles, CA 90095, USA; c Jonsson Comprehensive Cancer Center, University of California Los Angeles, Los Angeles, CA 90095, USA; d Parker Institute for Cancer Immunotherapy UCLA, USA; e David Geffen School of Medicine at UCLA, University of California Los Angeles, Los Angeles, CA 90095, USA

**Keywords:** Immunosuppressive tumor microenvironment, Immunotherapy resistance, Trap antibody, Bispecific antibody, Angiogenesis, Wound healing, Fibrosis, Combination immunotherapy

## Abstract

Immune checkpoint blockade (ICB) has become well-known in cancer therapy, strengthening the body’s antitumor immune response rather than directly targeting cancer cells. Therapies targeting immune inhibitory checkpoints, such as PD-1, PD-L1, and CTLA-4, have resulted in impressive clinical responses across different types of solid tumors. However, as with other types of cancer treatments, ICB-based immunotherapy is hampered by both innate and acquired drug resistance. We previously reported the enrichment of gene signatures associated with wound healing, epithelial-to-mesenchymal, and angiogenesis processes in the tumors of patients with innate resistance to PD-1 checkpoint antibody therapy; we termed these the Innate Anti-PD-1 Resistance Signatures (IPRES). The TGF-β and VEGFA pathways emerge as the dominant drivers of IPRES-associated processes. Here, we review these pathways’ functions, their roles in immunosuppression, and the currently available therapies that target them. We also discuss recent developments in the targeting of TGF-β using a specific antibody class termed trap antibody. The application of trap antibodies opens the promise of localized targeting of the TGF-β and VEGFA pathways within the tumor microenvironment. Such specificity may offer an enhanced therapeutic window that enables suppression of the IPRES processes in the tumor microenvironment while sparing the normal homeostatic functions of TGF-β and VEGFA in healthy tissues.

## 1. Introduction

Since a healthy immune system is innately able to suppress tumors, it has been suggested that cancer is synonymous to immune dysfunction (Zappasodi, Merghoub, & Wolchok, 2018). Thus, reinvigorating tumor-specific immune response is a promising way to control and cure cancer. The remarkable clinical results of blocking immune inhibitory checkpoints such as programmed cell death protein (PD-1), programmed cell death-ligand 1 (PD-L1), and cytotoxic T-lymphocyte antigen-4 (CTLA-4) in some cancers during the last decade have propelled immune checkpoint blockade (ICB)-based immunotherapy into popularity. Approximately one third of patients with advanced metastatic melanoma responded to ICB using monoclonal antibody (mAb) against PD-1 (anti-PD-1) (Hamid et al., 2013; Robert et al., 2015, 2015; Weber et al., 2015). Subsequently, the combination of anti-PD-1 and anti-CTLA-4 was approved as a first line therapy for the treatment of patients with unresectable or metastatic melanoma (Larkin et al., 2015; Postow et al., 2015). The combination therapy regimen achieved an objective response rate of 59%, but it was accompanied by a high frequency of grade 3/4 treatment-related adverse events (trAEs) caused by a hyperactivated immune system (Johnson, Nebhan, Moslehi, & Balko, 2022; Larkin et al., 2015; Postow et al., 2015).

Besides demonstrating efficacy in melanoma, anti-PD-1, anti-PD-L1 and/or anti-CTLA-4 has been integrated as part of standard therapy in cancers such Hodgkin’s lymphoma, renal cell carcinoma (RCC), and non-small cell lung cancer (NSCLC) (Wu et al., 2019; Yarchoan, Hopkins, & Jaffee, 2017; Zhao, Zhao, & Zhao, 2020). Other common malignancies such as bladder and breast cancers also respond to ICB mono-therapies at a rate of around 10–20% (Tabana, Okoye, Siraki, Elahi, & Barakat, 2021; van der Heijden et al., 2021; Zhao et al., 2020). However, even for cancer types with higher rates of response to ICB such as melanoma, a significant fraction of patients’ tumors is either innately resistant or eventually acquires resistance to the therapy. Various mechanisms behind differing responses to ICB-based immunotherapy have been discussed in multiple excellent reviews (Bruni, Angell, & Galon, 2020; Bu, Mahoney, & Freeman, 2016; Jenkins, Barbie, & Flaherty, 2018; Kalbasi & Ribas, 2020; Schoenfeld & Hellmann, 2020; Sharma, Hu-Lieskovan, Wargo, & Ribas, 2017). Briefly, these mechanisms can be generally classified into two categories: tumor-intrinsic and tumor-extrinsic. Tumor-intrinsic mechanisms include impaired tumor antigen presentation and loss of interferon sensitivity through loss-of-function alterations in the JAK/STAT signaling pathway (Gao et al., 2016; Garcia-Diaz et al., 2017; Gettinger et al., 2017; Grasso et al., 2018, 2020; Kalbasi et al., 2020; Manguso et al., 2017; Pan et al., 2018; Patel et al., 2017; Sade-Feldman et al., 2017; Shin et al., 2017; Zaretsky et al., 2016). Examples of tumor-extrinsic mechanisms include activation of immunosuppressive immune and stromal cell populations such as myeloid derived suppressor cells (MDSCs), M2-like macrophages, immature DCs, regulatory T cells (Tregs) and cancer associated fibroblasts (CAFs) (Binnewies et al., 2018; Dudek, Martin, Garg, & Agostinis, 2013; Feig et al., 2013; Gabrilovich & Nagaraj, 2009; Hamid et al., 2013; Joyce & Fearon, 2015; Rahma & Hodi, 2019). These cell populations are known to be involved in wound healing—a process during which the body attenuates initial inflammation at a site of injury to enable tissue repair. The hallmarks of wound healing also share significant similarities with those of cancer in general (MacCarthy-Morrogh & Martin, 2020). Our group and others have reported wound healing-related transcriptional signatures associated with T cell suppression and ICB resistance in melanoma, gastric, bladder, urothelial and microsatellite stable colorectal cancer (Bagaev et al., 2021; Cui et al., 2021; Hugo et al., 2016; Kim et al., 2019; Zeng et al., 2019, 2021). This set of gene expression signatures, termed the innate anti-PD-1 resistance signatures (IPRES), were highly expressed in the pre-treatment tumors of patients who did not benefit from anti-PD-1 therapy (Hugo et al., 2016). Subsequent analyses showed that the combination of the interferon pathway activity and IPRES-related immunosuppressive stromal scores are accurate predictors of ICB response in melanoma, gastric and metastatic urothelial carcinoma (Cui et al., 2021; Jiang et al., 2018; Zeng et al., 2021). The biological processes under IPRES were dominated by angiogenesis, hypoxia, epithelial to mesenchymal transition (EMT), and extracellular matrix remodeling, all of which are immunosuppressive processes related to the TGF-β and VEGFA pathways (Bu et al., 2016; MacCarthy-Morrogh & Martin, 2020; Parayath, Padmakumar, Nair, Menon, & Amiji, 2020; Schäfer & Werner, 2008). Therapeutic agents targeting TGF-β and VEGFA, the representative pathways of IPRES, may synergize with existing immunotherapies to overcome ICB resistance.

This review summarizes existing literatures on recent strategies that combine ICB with therapeutics targeting the TGF-β and VEGFA pathways. Of note, we discuss the potential of “trap” antibodies, a class of bispecific antibodies capable of binding to two distinct proteins, to enhance the therapeutic windows of TGF-β and VEGFA pathway inhibition in the context of improving ICB response.

## 2. The activities of TGF-β and VEGFA pathways associate with worse prognosis across cancers

Analysis of pan-cancer TCGA data revealed four distinct types of tumor microenvironments (TMEs): 1) immune-enriched, fibrotic; 2) immune-enriched, non-fibrotic; 3) fibrotic; and 4) immune-desert (Bagaev et al., 2021). An immune enriched microenvironment displays high enrichment of gene signatures associated with immune cells such as T, NK, and B cells, which are associated with antitumor immune response, and macrophages, neutrophils, MDSCs, and regulatory T cells (Tregs), which are associated with pro-tumor, immunosuppressive processes. The fibrotic TME shows significant enrichment of cancer associated fibroblasts (CAF), angiogenesis, and extracellular matrix remodeling traits, which overlap significantly with known wound healing processes and IPRES. Bagaev et al. discovered that immune-enriched, non-fibrotic TMES benefited the most from immunotherapy while both fibrotic and immune-depleted TMEs strongly correlated with worsened patient prognoses after ICB treatment in melanoma, bladder, and gastric cancers. Consequently, the authors suggest combining ICB with stromal signaling inhibition, potentially in the form of anti-TGF-β, anti-VEGFA or anti-VEGFR antibodies/small molecule inhibitors, for patients with fibrotic TMEs (Bagaev et al., 2021).

In line with the role of the TGF-β pathway in dampening antitumor immune response, Jiang et al. showed that *TGF-β1* transcript levels are significantly correlated with T cell dysfunction only in melanoma displaying high cytotoxic T cell (CTL) infiltration (Jiang et al., 2018). This report also highlighted a general anti-correlation between the levels of CTL and immunosuppressive immune populations such as M2-like, tumor-associated macrophages (TAMs), MDSCs, and CAFs. In microsatellite stable colorectal cancer (MSS CRC), both wound healing signatures and *VEGFA* mRNA expression correlated with later disease stage (Kim et al., 2019). Instead of wound healing signatures, the microsatellite instability-high colorectal tumors (MSI CRC) are enriched with interferon gamma (IFN-γ)-related gene signatures; higher IFN-γ and lower wound healing signature enrichments were proposed to be the drivers of the ICB response in MSI CRC but not MSS CRC.

Other studies also reported the correlation between the enrichment of stromal signatures and worsened prognosis in patients with melanoma, gastric, metastatic urothelial and colorectal cancer (Calon et al., 2015; Zeng et al., 2019, 2021). Zeng et al. devised a combined score of the TME (termed “TMEscore”), which considers the immune- and stromal-activation scores, to predict the overall survival of gastric cancer patients. Notably, TMEscore can predict response in ICB-treated melanoma, metastatic urothelial carcinoma (Zeng et al., 2019) and metastatic gastric cancer (Zeng et al., 2021). In a separate study, high expression of CAF and TGF-β signaling marker genes identify patients with poor-prognoses across CRC subtypes (Calon et al., 2015). Using tumor organoid models of human CRC that express high level of TGF-β, abrogation of TGF-β signaling was shown to significantly reduce tumor metastasis in mice.

Thus, multiple analyses of large cancer datasets have demonstrated a significant (anti) correlation between activities of TGF-β and VEGFA pathways and levels of antitumor immune response. The next logical question is whether these pathways are readily targetable, and if so, whether targeting them can improve the efficacy of existing ICB therapies.

## 3. The VEGF signaling pathway and its role in tumor angiogenesis

The VEGF protein family consists of proteins VEGFA, VEGFB, VEGFC, VEGFD, VEGFE (virally encoded), and proangiogenic molecule placental growth factor (PGF/PlGF) (Apte, Chen, & Ferrara, 2019; Carmeliet & Jain, 2011; Ellis & Hicklin, 2008; Ferrara & Adamis, 2016). VEGFA, an angiogenic protein frequently implicated in human disease, signals through binding with its main receptor VEGFR-2 (also known as KDR or Flk-1). A few splicing isoforms of VEGFA exist: VEGFA_121_, VEGFA_165_, VEGFA_189_ and VEGFA_206_. The shorter VEGFA_121_ isoform is highly diffusible, while the longer VEGFA_189_ and VEGFA_206_ are usually bound to the extracellular matrix (ECM) through their heparin binding domains (Cross, Dixelius, Matsumoto, & Claesson-Welsh, 2003; Ferrara, Gerber, & LeCouter, 2003; Olsson, Dimberg, Kreuger, & Claesson-Welsh, 2006). VEGFA_165_ displays a more intermediate characteristic between being ECM-bound and freely diffusible. It is the main functional isoform expressed in normal tissues and tumors (Olsson et al., 2006; Stalmans et al., 2002). Heparin binding VEGFA isoforms also bind the neuropilin 1 (NRP1) co-receptor, which stabilizes and enhances VEGFA-VEGFR-2 interaction (Olsson et al., 2006; Soker, Takashima, Miao, Neufeld, & Klagsbrun, 1998).

VEGFR-2 is known to be expressed on endothelial cells in the tumor vasculature. It is the main mediator of VEGFA-induced angiogenesis and modulation of vascular permeability (Cross et al., 2003; Ferrara & Adamis, 2016; Olsson et al., 2006; Sakurai, Ohgimoto, Kataoka, Yoshida, & Shibuya, 2005; Terman et al., 1992; Wang, Bove, Simone, & Ma, 2020). VEGFA binding to VEGFR-2 induces receptor dimerization and trans-autophosphorylation of multiple tyrosine residues on the cytoplasmic tail of the receptor. Phosphorylation of the Tyr1175 residue has been shown to be critical in the activation of multiple downstream signaling cascades such as PLCγ-PKC-MAPK, PLCγ-PKC-eNOS, SHB-PI3K-Akt, SHB-FAK-paxillin, and NCK-p38-MAPKAPK2/3, which are crucial in the proliferation, survival and migration of endothelial cells and angiogenesis in general (Sakurai et al., 2005; Shibuya, 2011; Wong & Jin, 2005). In parallel, the phosphorylation of Tyr951 induces the binding of TSAd and Src proteins, which subsequently activates VE cadherin-mediated regulation of vascular permeability (Li et al., 2016; Sun et al., 2012). For a comprehensive review of VEGFR-2 signaling, see (Wang et al., 2020).

VEGFA, along with VEGFB and PlGF, also binds the VEGFR-1 receptor. Interestingly, while VEGFA binds more strongly to VEGFR-1 (Flt-1), the lack of independent mitogenic or angiogenic effect of the VEGFA-VEGFR-1 interaction suggests that VEGFR-1 may function as a negative regulator of VEGFR-2 activation (Park, Chen, Winer, Houck, & Ferrara, 1994). VEGFB signaling through VEGFR-1 does not have a direct effect on the proliferation and survival of endothelial cells but is required for the development of normal heart vasculature and recovery from heart ischemia (Bellomo et al., 2000). On the other hand, PlGF binding to VEGFR-1 can either directly induce angiogenic processes via Akt pathway activation or indirectly enhance the VEGFA-VEGFR-2 pathway by occupying VEGFR-1 (Autiero, Luttun, Tjwa, & Carmeliet, 2003; Fischer, Mazzone, Jonckx, & Carmeliet, 2008). PlGF pathway activation not only induces vascular development and maintenance in healthy tissues but also acts as an angiogenic switch in cancer (Fischer et al., 2008). The other VEGF proteins, VEGFC and VEGFD, are implicated in the regulation of lymphoangiogenesis through their specific binding to VEGFR-3 (Alitalo, Tammela, & Petrova, 2005; Karkkainen et al., 2004).

## 4. Targeting VEGFA improves antitumor immunity

The expression of VEGFA in the tumor and TME is associated with increased tumor microvessel density, invasiveness, metastasis, and worsened patient prognosis (Apte et al., 2019; Butler, Kobayashi, & Rafii, 2010; Ferrara & Adamis, 2016; Jayson, Kerbel, Ellis, & Harris, 2016; Kerbel, 2008). VEGFA stimulates the proliferation of endothelial cells, forming a structurally abnormal and leaky tumor vasculature (Baluk, Hashizume, & McDonald, 2005; Ferrara, 2021; Jain, 2003, 2005; Nagy, Chang, Dvorak, & Dvorak, 2009). This results in high interstitial fluid pressure and collapsed intratumoral vasculature that hinders efficient blood flow and immune cell trafficking into the tumor. Beyond its role in tumor angiogenesis, VEGFA is also involved in immunomodulation within the TME (Apte et al., 2019; De Palma, Biziato, & Petrova, 2017; Elamin, Rafee, Toomey, & Hennessy, 2015; Fukumura, Kloepper, Amoozgar, Duda, & Jain, 2018; Huang et al., 2018; Lee, Yang, Chon, & Kim, 2020; Motz & Coukos, 2011). Tumor-derived VEGFA, along with other pro-angiogenic factors, can recruit and activate immune and stromal cell populations that are involved in physiological wound healing; they are recruited to “heal” the tumor. VEGFA binding to VEGFR-1+ monocytes and macrophages can induce their migration into the TME (Barleon et al., 1996). Alternatively activated (“M2-like”) TAMs, MDSCs, and tumor associated neutrophils (TANs) collectively produce pro-angiogenic growth factors (e.g., VEGFA, PlGF, EGF, FGF family, PDGF-β, TGF-β and Ang-2) and immunosuppressive cytokine/chemokines (e.g., IL-6, IL-8, IL-10 and CXCL12) (Fukumura et al., 2018; Huang et al., 2018; Lee et al., 2020; Liang & Ferrara, 2016; Maenhout, Thielemans, & Aerts, 2014; Murdoch, Muthana, Coffelt, & Lewis, 2008; Nagarsheth, Wicha, & Zou, 2017; Ozel et al., 2022). VEGFA has also been proposed to recruit immune-suppressive regulatory T cells (Tregs) into the TME (Facciabene et al., 2011; Goel & Mercurio, 2013; Huang et al., 2018; Khan & Kerbel, 2018).

The abundance and antitumor activity of cytotoxic T cells (CTLs) are negatively regulated by VEGFA through direct binding to VEGFR-2 expressed on these T cells (Gavalas et al., 2012; Huang et al., 2007; Ohm et al., 2003). Gavalas et al. showed the expression of VEGFR-2 on activated CTLs. These CTLs displayed a diminished proliferation rate and cytotoxicity when exposed to VEGFA (Gavalas et al., 2012). VEGFA has also been reported to upregulate the expression of Fas ligand (FasL/CD95L) of the tumor vasculature, which specifically induces apoptosis of CTLs but not Tregs (Motz et al., 2014). The maturation of dendritic cell (DC) and antigen presentation capability of mature DCs are also negatively impacted by VEGFA, thereby limiting tumor specific T cell priming (Elamin et al., 2015; Gabrilovich et al., 1996; Huang et al., 2007; Khan & Kerbel, 2018; Mimura, Kono, Takahashi, Kawaguchi, & Fujii, 2007; Oyama et al., 1998). Importantly, VEGFA upregulates TOX expression in CD8+ T cells, initiates TOX mediated transcriptional re-programming that promotes T cell exhaustion, and upregulates multiple checkpoint inhibitor receptors such as PD-1, LAG-3, TIM-3 and TIGIT on these T cells (Kim et al., 2019).

Existing strategies to target the VEGF-VEGFR pathway can be categorized into 1) antibody or antibody-like therapeutics that prevent the binding of VEGF ligands to the VEGFR (e.g., bevacizumab and ranibizumab, which bind VEGFA, aflibercept (also known as “VEGF-trap”), which binds VEGFA/B and PlGF, and ramucirumab, which binds VEGFR-2) and 2) small molecule tyrosine kinase inhibitors (TKIs) against VEGFR1–3 (e.g., sorafenib, sunitinib, pazopanib, cabozantinib, lenvatinib); these TKIs can also target the kinase domain of related receptor tyrosine kinases such as PDGFRa/b, FGFR1–3, c-KIT, and RET (Apte et al., 2019; Ferrara & Adamis, 2016; Garcia et al., 2020; Jayson et al., 2016; Olsson et al., 2006; Zirlik & Duyster, 2018). These VEGF-targeting agents have been tested in multiple cancer types as single agents or in combination with other therapies (reviewed in (Ferrara & Adamis, 2016; Fukumura et al., 2018; Jain, 2014; Jayson et al., 2016; Khan & Kerbel, 2018; Lee et al., 2020; Zirlik & Duyster, 2018)). Given its generally immunosuppressive role and specific effects on T cell checkpoint expression, VEGFA has been targeted in combination with ICB in many studies over the past six years (Fukumura et al., 2018; Khan & Kerbel, 2018; Lee et al., 2020).

Table 1 lists the combinations of anti-VEGF and ICB agents targeting the PD-1/PD-L1 axis which have been approved by the FDA or have completed phase III studies. Several combinations of immune checkpoint blockade targeting PD1/PD-L1 and bevacizumab (anti-VEGFA) or VEGFR2-targeting TKIs have been FDA-approved to treat the highly vascularized RCC (Choueiri et al., 2020, 2021; Motzer et al., 2019, 2021, 2022; Powles et al., 2020; Rini et al., 2019; Rini, Powles, et al., 2019) and hepatocellular carcinoma (HCC) (Cheng et al., 2022; Finn et al., 2020). In addition, two separate combinations were approved for two gynecological cancers: the microsatellite stable endometrial (Makker et al., 2019; Marth et al., 2022) and PD-L1 positive cervical cancers; these cancers respond well to the combination of PD-1 and VEGF pathway inhibition in combination with chemotherapy (Rubinstein & Makker, 2020). Two recently concluded phase III studies also showed some efficacy of combining bevacizumab, nivolumab, and chemotherapy in non-squamous, NSCLC (Sugawara et al., 2021) and metastatic colorectal carcinoma (mCRC) (Lenz et al., 2022).

The FDA-approved VEGF-trap, aflibercept, is indicated for mCRC (Stewart, 2011). Of note, the combination of aflibercept and pembrolizumab displayed an acceptable safety profile with antitumor activity in a phase 1 study on patients with melanoma, RCC, and mesothelioma (Tyan et al., 2021). In general, the combination of VEGFA targeting and ICB has an acceptable safety profile that is comparable to that of the standard of care. As such, we expect more clinical trials testing the combination of VEGFA pathway inhibition and ICB in more diverse cancer types, especially those on which ICB alone is less efficacious.

## 5. The history of targeting the TGF-β pathway

TGF-β, or Transforming Growth Factor Beta, is a ubiquitous cytokine that is active in various processes within the mammalian cell. It can inhibit cell proliferation and promote differentiation, consistent with its role in maintaining tissue homeostasis and suppressing aberrant neo-plastic growth (Morikawa, Derynck, & Miyazono, 2016; Seoane, 2006). Curiously, TGF-β switches from demonstrating tumor-suppressing properties in early stage tumors to tumor-promoting properties in late stage tumors (Lebrun, 2012; Massagué, 2008; Padua & Massagué, 2009; Papageorgis, 2015; Principe et al., 2014; Seoane & Gomis, 2017; Tian & Schiemann, 2009); this phenomenon is termed the “TGF-β paradox”. Such pleiotropic, even contradictory, roles of TGF-β have complicated efforts to suppress cancer growth through the modulation of this pathway.

The TGF-β ligand has three isoforms: TGF-β1, -β2, and -β3. Each starts as an inactive precursor protein containing a signal peptide, a latency-associated polypeptide (LAP), and the mature C-terminal polypeptide (Hinck, Mueller, & Springer, 2016; Morikawa et al., 2016; Moses, Roberts, & Derynck, 2016). Two precursor proteins subsequently dimerize through the formation of a disulfide bond across the mature polypeptide region. The N-terminal LAP is proteolytically cleaved by furin but stays non-covalently associated with the TGF-β dimer. This complex (termed the small latent complex) can associate through disulfide bonding with latent TGF-β binding protein (LTBP) into a large latent complex (LLC) that is bound to ECM proteins such as collagen, thrombospondin and fibronectin. The small latent complex can also bind glycoprotein-A repetitions predominant (GARP) proteins on the plasma membrane. These arrangements allow the deposition of TGF-β ligands that can only initiate the downstream signaling after an activation-driven cleavage from the ECM/LTBP (hence their “latent” characteristic) (Robertson & Rifkin, 2016).

Knockout mouse studies for the three TGF-β isoforms have been used to further elucidate their specific roles. TGF-β1 is important for hematopoiesis and vascular development (Dickson et al., 1995). Additionally, TGF-β1 expression and activation are rapidly upregulated in response to injury, and are crucial for efficient wound healing *in vivo* (Kane, Hebda, Mansbridge, & Hanawalt, 1991; Sporn et al., 1983). TGF-β2 contributes to development of the skeleton, heart, eyes, ears, and urogenital tract (Sanford et al., 1997). TGF-β3 is necessary for the development of the pulmonary system where a deficit leads to cleft palates and death (Proetzel et al., 1995). In addition, mice deficient in TGF-β2 and -β3 expression reveal defects in their central nervous system (Vogel, Ahrens, Büttner, & Krieglstein, 2010).

There are multiple excellent reviews covering the details of the TGF-β family proteins and their related signaling pathways (Derynck & Budi, 2019; Derynck & Zhang, 2003; Glasgow & Mishra, 2008; Haque & Morris, 2017; Massagué, Blain, & Lo, 2000; Morikawa et al., 2016; Shi & Massagué, 2003; Smith, Robin, & Ford, 2012). Briefly, TGF-β signaling is initiated by TGF-β ligand binding to TGF-β receptor-2 (TβRII), a trans-membrane serine-threonine kinase. Next, facilitated by TβRIII, TGF-β ligand binding induces a conformational change in TβRII and recruits TβRI, which subsequently leads to cross-phosphorylation of activation of TβRI. Then, receptor-regulated Smad proteins (Rsmad), Smad2 or Smad3, are recruited to TβRI and phosphorylated. Phosphorylated Smad2 or Smad3 forms a heterodimeric complex with Smad4 (a co-Smad) and enters the nucleus where it works with other cofactors to bind DNA and modulate the TGF-β pathway’s downstream gene expression. TGF-β-Smad pathway activation generally regulates cell proliferation and, in some contexts, induces cell differentiation to maintain tissue homeostasis (Kubiczkova, Sedlarikova, Hajek, & Sevcikova, 2012). In addition to Smad-dependent downstream processes, TGF-β can also activate ERK, PI3K/Akt, NF-κB, the small GTPases Rac/Cdc42, JNK, and p38 MAPK pathways (Bakin, Rinehart, Tomlinson, & Arteaga, 2002; Bakin, Tomlinson, Bhowmick, Moses, & Arteaga, 2000; Derynck & Zhang, 2003; Lee et al., 2007; Mu, Gudey, & Landström, 2012; Sorrentino et al., 2008). The activation of TGF-β signaling also upregulates Smad6 and Smad7, which can inhibit ligand-induced R-Smad activation by directly binding to TβRI at its cytoplasmic tail. This negative feedback loop prevents continuous activation of the TGF-β signaling pathway (Miyazawa & Miyazono, 2017).

As cancer progresses, tumor cells stop responding to TGF-β-mediated growth inhibition, potentially through somatic mutations. Of note, mutations in TβRII are common in colon, pancreatic, lung, and brain cancers, while TβRI mutations are less frequent (Levy & Hill, 2006; Massagué, 2008; Meulmeester & ten Dijke, 2011). TGF-β overexpression has been clinically observed in various cancers, including malignant melanoma, breast, colon, esophagus, stomach, liver, lung, kidney, pancreas, prostate, and brain (Haque & Morris, 2017). Tumor cells also upregulate TGF-β expression to stimulate EMT (which is involved in cancer invasion and metastasis) (Galliher & Schiemann, 2007; Sánchez-Elsner et al., 2001; Yuan et al., 2014), angiogenesis (Goumans, Liu, & ten Dijke, 2009; Nishida, Yano, Nishida, Kamura, & Kojiro, 2006; Sánchez-Elsner et al., 2001), and immunosuppression (Batlle & Massagué, 2019; Chakravarthy, Khan, Bensler, Bose, & De Carvalho, 2018; Derynck, Turley, & Akhurst, 2021; Jiang et al., 2018; Mariathasan et al., 2018; Tauriello et al., 2018). Intriguingly, like its effect on tumor cell proliferation, TGF-β also has a paradoxical effect on angiogenesis; low levels of TGF-β promote angiogenesis by increasing the proliferation of endothelial cells and VEGFA expression, while high TGF-β levels hinders angiogenesis (Madri, Pratt, & Tucker, 1988; Pertovaara et al., 1994).

In addition to inducing pro-tumorigenic angiogenesis and EMT, TGF-β directly affects various immune cell populations (reviewed in (Batlle & Massagué, 2019; Derynck et al., 2021)). Exogenous TGF-β was shown to inhibit Th1 and cytotoxic T cell differentiation and activity (Batlle & Massagué, 2019; Oh & Li, 2013; Sad & Mosmann, 1994; Sledzińska et al., 2013). *In vivo* studies demonstrated that TβRII-deficient CD4+ and CD8+ T cells displayed stronger TCR activation and effector functions in the presence of a weak antigen (Sledzińska et al., 2013). Additionally, TGF-β induces the expression of *FOXP3*, which is the master regulator of CD4+ Treg differentiation (Chen et al., 2003; Fantini et al., 2004; Strainic, Shevach, An, Lin, & Medof, 2013). Furthermore, TGF-β can interfere with cytotoxic NK cell (Laouar, Sutterwala, Gorelik, & Flavell, 2005; Yu et al., 2006) and DC (Nandan & Reiner, 1997; Papaspyridonos et al., 2015) functions. In the myeloid compartment, TGF-β skews the polarization of macrophage and neutrophils into a phenotype that is more pro-tumorigenic and related to wound healing (Fridlender et al., 2009; Li, Han, Guo, Zhang, & Cao, 2009; Mantovani, Sozzani, Locati, Allavena, & Sica, 2002; Standiford et al., 2011).

In experimental models, the TGF-β pathway has been successfully blocked through multiple strategies: 1) antisense oligonucleotide molecules that directly inhibit TGF-β synthesis (e.g. Trabedersen, AP 11014); 2) monoclonal antibodies (e.g. metelimumab, lerdelimumab, fresolimumab), 3) TGF-β decoys that sequester the TGF-β ligand from binding to the receptor (e.g. AVID200, SRK-181); 4) small molecule inhibitors that interfere with the activation of downstream Smad proteins (e.g. galunisertib and vactosertib). The mechanism of action and clinical testing of these agents (alone and in combination with existing therapies) have been extensively reviewed (Ciardiello, Elez, Tabernero, & Seoane, 2020; Derynck et al., 2021; Haque & Morris, 2017; Lee, 2020). Overall, the clinical testing of TGF-β pathway inhibitors have had limited success and have not resulted in FDA approval. Given the independent and complementary immunosuppressive functions of immune inhibitory checkpoints (e.g., PD-1, PD-L1, or CTLA4) and the TGF-β pathway (Derynck et al., 2021; Lind et al., 2020; Strauss et al., 2018), combined inhibition (i.e., ICB plus TGF-β targeting agents) holds significant promise as an effective therapeutic strategy. Indeed, multiple ongoing clinical trials are evaluating the efficacy of combinatorically targeting TGF-β and PD-1/PD-L1 (Table 2).

## 6. Trap antibodies: localized targeting of TGF-β

**Fig. 1.**
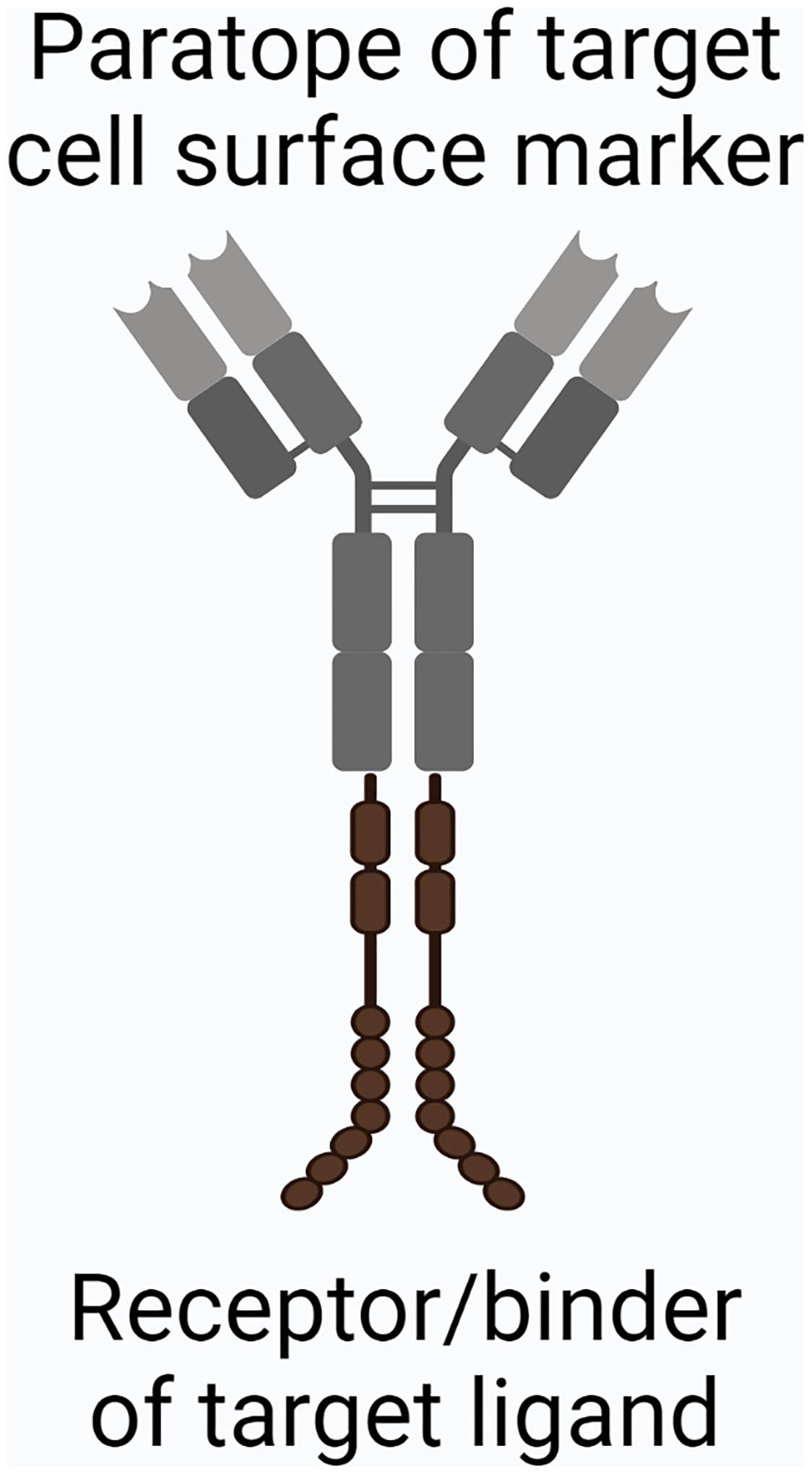
The general structure of a trap antibody. Schematic of a generic trap antibody structure. The variable regions/F_ab_ (shown in gray) are specific for a cell-surface protein marker on a target cell population. The constant region/F_c_ (shown in brown) is fused to either an antibody or ligand binding domain of the ligand to be “trapped”, thereby acting as a mimic to the actual receptor of the molecule.

Given TGF-β’s critical function in maintaining immune homeostasis (Horwitz, Fahmy, Piccirillo, & La Cava, 2019; Sanjabi, Oh, & Li, 2017), systemic targeting of the TGF-β pathway can result in serious adverse events such as cardiovascular inflammation (Colak & ten Dijke, 2017; Teixeira, ten Dijke, & Zhu, 2020). Thus, therapies targeting TGF-β need to be localized to the tumor site and/or specific cell populations associated with TGF-β ligands. An antibody-ligand “trap”, a class of bispecific antibodies, can accomplish this localization goal (Ravi et al., 2018). The constant region of the bispecific antibody binds the target ligand, while the variable domains of the antibody bind to a specific cell surface marker; this antibody effectively “traps” the target ligand near the target cell. In short, we refer to this type of antibody as “trap antibody” (see Fig. 1). When many trap antibodies bind their target marker on cell surfaces, they can efficiently sequester the target ligands near target cells by virtue of their high local concentration. This mechanism of action results in a localized, cell type-specific reduction of the unbound ligand around and subsequent suppression of pathway activation by the ligand within the target cell population.

For instance, 4T-Trap is a trap antibody that traps TGF-β ligands while binding to CD4 receptors on T cells (Li et al., 2020). 4T-Trap is engineered by adding TβRII’s extracellular domain to the constant region of ibalizumab (a non-immunosuppressive CD4 antibody). 4T-Trap was designed based on the observation that loss of TβRII in CD4 + T cells but not CD8+ T cells suppressed the growth of PyMT (a mouse model of breast cancer) and MC38 (colorectal cancer mouse model) (Liu et al., 2020). Specifically, Liu et al. reported that the antitumor effect of TβRII loss was mediated by enhanced Th2 differentiation and interleukin-4 (IL-4) cytokine expression by CD4+ T cells. The activation of Th2 T cells renormalized tumor vasculature, causing cancer cell hypoxia and death. Notably, in both models, antitumor response driven by TβRII loss is fully dependent on the Th2 cytokine, IL-4.

**Fig. 2.**
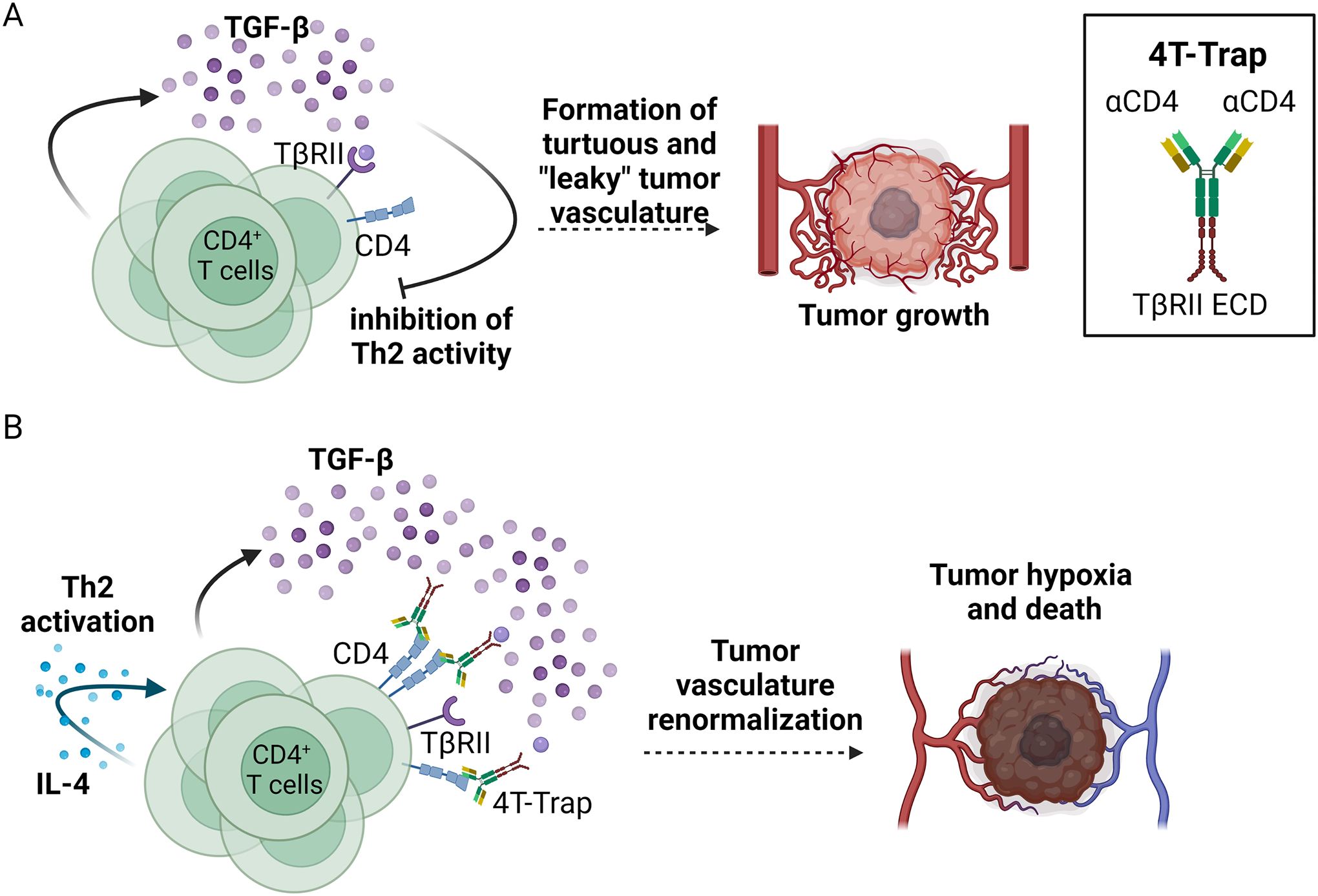
4T-Trap’s proposed mechanism of action. **A.** Activated CD4+ T cells secrete TGF-β1 and induce a suppressive, autocrine TGF-β signaling through TβRII. The activation of TGF-β pathway in the CD4+ T cells prevents them from to efficiently differentiating into T helper type 2 cells (Th2 cells), which leads to the formation of “leaky” tumor vasculatures and tumor growth. **B.** The F_ab_ regions of 4T-Trap bind the CD4 receptor on T cells while the extracellular domain (ECD) of TβRII on the F_c_ region of 4T-Trap binds to TGF-β ligands in the CD4+ T cell locale. The trapping of TGF-β ligands prevents their binding to the TβRII receptor on the CD4+ T cells. Decreased TGF-β signaling promotes the differentiation of the CD4+ T cells into IL-4 secreting, Th2 T cells. Fully functional Th2 CD4+ T cells then induce the normalization of the tumor vasculature, which leads to tumor cell hypoxia and death.

Li et al. utilized 4T-Trap to mimic the specific deletion of TβRII in CD4 + T cells (Li et al., 2020). When they compared 4T-Trap to a non-targeted TGF-β-trap, they observed that only 4T-Trap recapitulated the tumor vascular normalization and IL-4 induction in TβRII-deficient CD4+ T cells. 4T-Trap treatment subsequently induced hypoxia-driven tumor cell death in mice with PyMT and MC38 tumors. Of note, the authors suggested that one of the major sources of the TGF-β1 ligand were the activated CD4+ T cells themselves (i.e., autocrine TGF-β signaling). Thus, the efficacy of 4T-Trap may also be attributed to its ability to efficiently sequester (and internalize) TGF-β1 ligands as they are being secreted by activated CD4+ T cells (for an illustration of the mechanism of action of 4T-Trap, see Fig. 2). Furthermore, the tumor draining lymph nodes (tdLN) of 4T-Trap treated mice were enriched in effector memory CD4+ T cells, thereby demonstrating showing a suppressed TGF-β pathway activity. This observation suggests the ability of 4T-Trap to activate antitumor CD4+ T cells outside the TME. The induction of tumor hypoxia by 4T-Trap upregulated VEGFA expression, which motivated the authors’ targeting of both the TGF-β and VEGFA pathways in PyMT and MC38 tumor models. Indeed, co-administration of 4T-Trap and a VEGF-trap (modeled after the human VEGF-trap, aflibercept) synergistically suppressed tumor growth and prolonged mice survival (Li et al., 2020). This result strongly supports the notion of dual targeting of TGF-β and VEGFA (i.e., simultaneous targeting of IPRES processes) to achieve stronger antitumor activity than targeting either pathway alone.

Besides 4T-Trap, other effective preclinical and clinical trap examples, primarily combining TGF-β targeting agents with ICB, have been reported in recent years. For instance, Ravi et al. showcased the superior antitumor efficacy of two TGF-β trap antibodies, which were engineered from FDA-approved antibodies targeting CTLA-4 (ipilimumab) or PD-L1 (atezolizumab and avelumab) immune checkpoints (Ravi et al., 2018). For brevity, we will refer to these trap antibodies as CTLA4-TβRII trap and PDL1-TβRII trap, respectively. Using melanoma and triple negative breast cancer (TNBC) human cancer cell lines xenografted into NSG mice that were immune reconstituted using HLA-matched human bone marrow cells, Ravi et al. reported enhanced antitumor activity of CTLA4-TβRII trap over anti-CTLA4 monotherapy, a non-specific TGF-β-trap, as well as their combination. Tumors from mice treated with CTLA4-TβRII trap displayed higher proportions of 1) tumor reactive CD8+ IFNγ+ T cells, 2) CD4+ and CD8+ central memory T cells, and 3) lower percentage of FOXP3+ Tregs compared to control mice.

Because CTLA-4 is constitutively highly expressed in Tregs, and given Tregs’ dependence on the TGF-β pathway to maintain its activity (Chen et al., 2003; Tone et al., 2008), CTLA4-TβRII trap effectively prevented Treg differentiation and activity. CTLA4-TβRII trap also effectively suppressed the differentiation of CD4+ T helper cells to the Th17 lineage (inflammatory and autoimmune-related) since Th17 differentiation depends on IL-6 and TGF-β ligand. Strikingly, the authors observed that CTLA4-TβRII trap alone inhibits the growth of the TNBC tumor model better than a combination treatment using anti-CTLA-4 plus anti-PD1. The authors further reported the efficacy of PDL1-TβRII trap in suppressing tumor growth in the melanoma and TNBC models. The authors implicated that PDL1-TβRII functions by sequestering TGF-β near PD-L1-expressing tumor cells. As with CTLA4-TβRII, PDL1-TβRII reduced the proportion of intratumoral Tregs; the mechanism underlying this reduction was not described in detail. Since PD-L1 is not usually highly expressed on the surface of Tregs, it is possible that the localized sequestration of TGF-β in the TME indirectly limits the availability of unbound TGF-β ligand for Treg differentiation and activity.

Confirming the utility of sequestering TGF-β near PD-L1+ cell population, an independent study demonstrated the efficacy of M7824, a PDL1-TβRII trap (based on avelumab), in suppressing tumor growth and metastasis in orthotopic breast and colorectal cancer models (Lan et al., 2018). Importantly, M7824 conferred antitumor immunological memory that protected mice from tumor rechallenge long after treatment discontinuation. Combined treatment of M7824 with radiation therapy was shown to suppress the growth of not only the irradiated subcutaneous MC38 tumor but also the non-irradiated, opposite flank MC38 tumor. Such an abscopal effect, combined with a hint of immunological memory formation, strongly suggests that M7824 is capable of inducing a systemic, tumor-specific immune response. Unlike 4T-Trap, whose efficacy depends on CD4+ T cells, the authors showed that the antitumor activity of M7824 was dependent on cytotoxic CD8+ T cells and NK cells. In an *in vitro* study of M7824, Grenga et al. showed the ability of M7824 to modulate the immunogenicity of urothelial carcinoma cells, thus making them more susceptible to immune surveillance (Grenga et al., 2018). Specifically, the authors demonstrated that M7824 mediates NK cell-driven antibody-dependent cellular cytotoxicity against the tumor cells *in vitro*. Additionally, compared to anti-PD-L1 monotherapy, M7824 more strongly induced upregulation of intratumoral T-cell trafficking genes such as CXCL11 as well as bolstered antigen-specific cytotoxic T cell-mediated tumor cell lysis.

On the basis of favorable results from multiple preclinical studies, M7824 underwent a phase 1 clinical trial in a cohort of nineteen heavily pretreated patients with advanced solid tumors (Strauss et al., 2018). M7284 treatment led to one confirmed complete response in a patient with cervical cancer, near partial response in another patient with cervical cancer, and two durable confirmed partial responses in pancreatic and anal cancers. In two patients (with pancreatic cancer and carcinoid) who experienced progressing disease at the time of study entry, M7824 induced stable disease. Four of nineteen patients experienced grade three or higher adverse events such as skin infection secondary to localized bullous pemphigoid, anemia-associated colitis, and gastroparesis. Overall, M7824 seems to exhibit a manageable safety profile. Another phase I trial testing M7824 on patients with metastatic/locally advanced solid tumors in Asia (NCT02699515) also showed the clinical promise of M7824 (Bang et al., 2018). Combining the results of patients from original and expansion cohorts, 7 out of 31 heavily pretreated patients with advanced gastric cancer achieved an objective response (5 partial responses and 2 complete responses). Seven patients experienced grade 3–5 trAEs: anemia (2), diarrhea (1), abnormal hepatic function (1), rash (2) and 1 grade 5 AE (suspected rupture of pre-existing thoracic aortic aneurysm).

Despite the initial successes of M7824, it is important to note that several clinical studies were terminated early (see Table 2). One such example was a phase III study comparing the efficacy of M7824 as a first line treatment for patients with advanced, PD-L1 positive NSCLC. The comparator arm was pembrolizumab (the FDA-approved ICB for this cancer type). The interim analysis indicated that the trial was likely to miss its primary end point: progression free survival (PFS). We speculate that the TGF-β ligand’s main source/target cell population(s) in the NSCLC TME are likely not in the vicinity of PD-L1 expressing cell populations. Hence, this patient population could not leverage the bispecific merit of M7824. Other studies were terminated due to serious trAEs and/or tumor hyperprogression (Table 2). TGF-β signaling blockade has been associated with increased risk of bleeding, presumably caused by compromised vascular integrity. After all, TGF-β signaling on the pericytes is required for endothelial integrity (Derynck et al., 2021). Instances of tumor hyperprogression are of serious concern. In an inflamed TME with high PD-L1 expression, immune cell-derived TGF-β1 may suppress tumor proliferation; localized TGF-β blockade by M7824 may negate such suppression.

Nonetheless, additional studies on the immune, stromal, and tumor cell populations from the treatment-responding and -non-responding tumors are needed to dissect the mechanism of action (and non-action) of M7824. Such knowledge will be crucial to improve the design of future TGF-β-traps and to stratify patient populations that can benefit most optimally from M7824.

## 7. Discussion

Undermining IPRES by blocking the activity of its key pathways, VEGFA and TGF-β, has robust potential to improve clinical outcomes of patients with melanoma treated with ICB. Systemic targeting of VEGFA, along with its combination with ICB, are generally well tolerated in patients. The most frequent trAEs were hypertension or proteinuria, which were also commonly observed upon anti-VEGFA monotherapy and are generally manageable. As such, the combination of anti-VEGFA and ICB is being tested in a multiple tumor histologies. Thus far, the benefit of combined VEGFA and immune checkpoint inhibition is seen in tumors that respond to single agent anti-VEGFA therapy such as HCC, RCC, CRC, NSCLC, and gynecologic tumors (Table 1). One exception is glioblastoma, where the combination of anti-PD-1 and anti-VEGFA was not better than administering anti-VEGFA as a single agent (Reardon et al., 2018). In melanoma, improvements in overall survival by a single agent targeting VEGFA have historically been limited (Corrie et al., 2018). Nevertheless, several ongoing clinical trials are testing the combination of anti-VEGFA and anti-PD-1/PD-L1 in metastatic melanoma (NCT02681549, NCT04356729, NCT03175432).

More considerations should be factored into the design of strategies targeting the more pleiotropic TGF-β signaling. The treatment dosage and regimens of existing TGFβ inhibitors, anti-TGFβ antibodies or M7824 often have a relatively narrow therapeutic window as it is common for potent systemic inhibition of TGF-β signaling to confer substantial toxicities (Derynck et al., 2021). M7824, a PDL1-TβRII trap that binds TGF-β from the sites with high expression of PD-L1, has shown potential clinical efficacy in a phase 1 basket clinical trial of multiple solid tumor types (Strauss et al., 2018). It is worthwhile to note that a few of the subsequent clinical trials of M7824 were terminated or withdrawn, again due to safety concerns. It is possible that systemic T cell activation induced by the anti-PD-L1 portion of M7824 can also induce PD-L1 expression in other organs beyond the local TME. In such cases, localized sequestration of the TGF-β ligand near the (inflamed) PD-L1+ normal tissue will prevent the normal homeostatic response against such inflammation and result in immune-mediated toxicities. Although 4T-Trap is still in a preclinical stage, its merit of localized TGF-β inhibition in a specific antitumor CD4+ T cell population may result in potent antitumor effects as well as a more manageable toxicity profile (Li et al., 2020). Of note, the authors demonstrated that the combination of 4T-Trap and VEGF-trap, which targets two IPRES pathways, resulted in significantly stronger tumor control in mice. Since the combination of VEGF-trap with anti-PD-1 was found to be safe in patients with cancer (Tyan et al., 2021), a potential future combination regimen may involve the co-administration of 4T-Trap with VEGF-trap and ICB.

**Fig. 3.**
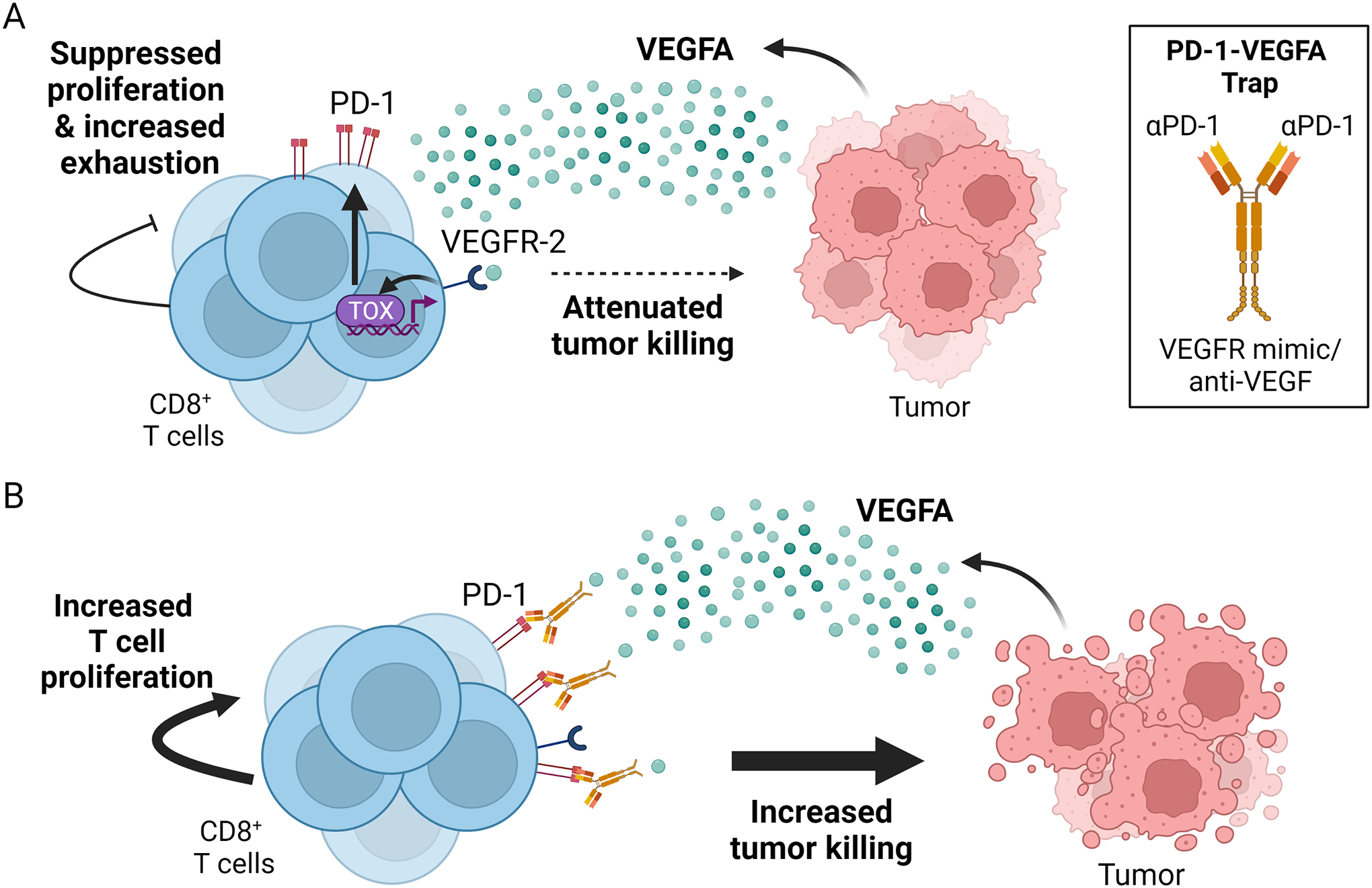
Co-targeting of PD-1 and VEGFA may relieve VEGFA-induced T cell exhaustion. **A.** Tumor and stroma-derived VEGFA binds to VEGFR-2 expressed on activated CD8+ T cells. The binding results in the activation of the master regulator of T cell exhaustion, TOX, which subsequently dials up the expression of multiple immune checkpoints on T cells. TOX activation can also suppress T cell proliferation and cytotoxic activities, resulting in attenuated tumor killing. **B.** The proposed mechanism of action of a PD1-VEGFA-trap, AK112. Upon binding to the PD-1 receptors expressed on activated CD8+ T cells, AK112 sequester VEGFAS protein in the nearby locale and decreases the activity of VEGFA/VEGFR-2 pathway signaling in T cells (while minimally impacting the effects of VEGFA signaling elsewhere). Reduced TOX activity relieves CD8+ T cells from exhaustion and dysfunction, which can ultimately lead to increased T cell-induced tumor killing.

Anti-VEGF therapies are associated with dose-limiting cardiovascular and non-cardiovascular toxicities despite their generally acceptable safety profiles (see http://www.uptodate.com/contents/toxicity-of-molecularly-targeted-antiangiogenicagents-cardiovascular-effects and http://www.uptodate.com/contents/toxicity-of-molecularly-targeted-antiangiogenicagents-non-cardiovascular-effects). Thus, in the same vein as the design of 4T-Trap, a cell type-specific VEGF-trap may also hold potential to enhance ICB efficacy in melanoma and other solid cancers. Given the specific inhibitory effects of VEGFA on tumor-reactive, cytotoxic CD8+ T cells (Gavalas et al., 2012; Kim et al., 2019), a VEGFA-trap directed to activated CD8+ T cells may improve antitumor T cell activities in VEGFA-rich TME. Indeed, VEGFA can induce the activation of the master regulator of T cell exhaustion, TOX, as well as the expression of the PD-1 checkpoint in tumor-reactive, CD8+ T cells (Kim et al., 2019). These observations motivate the design of a PD-1 directed, VEGFA trap antibody that binds specifically to PD-1+ T cells and protects them from VEGFA mediated suppression (a schematic of how PD-1-VEGFA-trap may function is illustrated in Fig. 3). One such PD-1-VEGFA-trap, AK112 (a humanized IgG1 bispecific anti-PD-1/VEGFA antibody), is currently being tested in multiple phase 2 clinical trials involving NSCLC, TNBC, and advanced gynecological tumors (NCT04736823, NCT05227664, NCT04870177). Results from a phase 1b trial of AK112 on patients with advanced/metastatic solid tumors that are refractory to standard therapies revealed a favorable safety profile and provided preliminary evidence of antitumor activity (Coward et al., 2021). While adverse events did occur in 55.2% of the patients, only three out of 29 patients experienced grade 3 trAEs, and no grade ≥ 4 AEs occurred. Of the 17 patients treated at doses ≥3 mg/kg once every two weeks, the objective response rate (ORR) was 23.5% (4/17) and disease control rate (DCR) was 64.7% (11/17). Given the fact that the tumors were highly refractory to existing therapies, this result highlights the potency of AK112. It remains to be seen if the response rate holds in the later phases of AK112 clinical testing.

It is possible that the application of AK112 or other cell surface marker-specific VEGF-Trap can induce intracrine VEGFA signaling. In this mode of signaling, the VEGFA protein activates the VEGFR-1 or VEGFR-2 receptor from within the cell (e.g., in the endoplasmic reticulum or the nucleus) (Wiszniak & Schwarz, 2021). Upon binding to the PD-1 receptor of CD8+ T cells, AK112-trapped VEGFA could dissociate from it in the acidic environment of the endosome, bind to VEGFR-2 and activate VEGFA signaling in the target T cells in an intracrine manner. Such a process could negatively affect T cells. Additional studies are needed to ascertain if 1) AK112 is internalized after binding to PD-1 and, 2) there is any evidence of intracrine VEGFR-2 phosphorylation in the T cells with AK112 treatment. In the case where intracrine VEGFA signaling is present, instead of using an antibody against VEGFA, one can utilize aflibercept, a recombinant VEGFR mimic, in the design of the PD-1-VEGFA-trap. The significantly higher binding affinity between aflibercept and VEGFA compared to VEGFR-2 and VEGFA should diminish the possibility of VEGFA dissociation from the trap antibody and subsequent binding to VEGFR-2 after PD-1 receptor internalization.

The efficacy of ICB in various cancers has informed a robust discussion between clinicians, scientists, and pharmaceutical stakeholders on optimal dosing and therapeutic regimens to maximize response rate, minimize toxicity, and improve survival of cancer patients. The application of novel trap antibodies against the immunosuppressive pathways represented by IPRES may uncover novel synergistic combinations with existing ICB-based immunotherapies. Such combinatorial treatments could optimally harness the immune system to suppress and eventually eradicate tumors in patients with cancer.

## Figures and Tables

**Table 1.** Completed phase III trials testing the combination of VEGF/VEGFR and PD-1/PD-L1 blocakade in solid tumors.

Drug combination	Tumor type	Clinical Trial ID	Phase	Status	Change in PFS (months)	Change in OS (months)	Frequency of grade 3–5 adverse events	References
Bevacizumab + Atezolizumab	Untreated Advanced or Metastatic Hepatocellular Carcinoma (HCC)	NCT03434379	FDA approved	Completed	2.6	5.8	45%	Finn et al., 2020, Cheng et al., 2022
Bevacizumab + Atezolizumab + Carboplatin + Paclitaxel	Untreated Metastatic Non-Squamous Non-Small Cell Lung Cancer (without EGFR and ALK mutation)	NCT02366143	FDA approved	Completed	1.5	4.5	58.5%	Socinski et al., 2018
Bevacizumab + Atezolizumab	Untreated Advanced Renal Cell Carcinoma (RCC)	NCT02420821	III	Completed	2.8# (ITT) 3.5# (PD-L1+)	0.8^ (ITT) 7.1^ (PD-L1+)	46%	Rini et al., 2019#, Motzer et al., 2022^
Bevacizumab + Pembrolizumab + platinum-based chemotherapy	Recurrent Metastatic or unresectable cervical cancer (PD-L1 combined positive score ≥ 1)	NCT03635567	FDA approved	Completed	2.2	>7.9 (ITT)	81.8%	Colombo et al., 2021
Bevacizumab + Nivolumab + Carboplatin + Paclitaxel	Untreated Metastatic Non-Squamous, Non-Small Cell Lung Cancer (without EGFR and ALK mutation)	NCT03117049	III	Active, not recruiting	4.0	0.7 (interim)	73.6%	Sugawara et al., 2021
Bevacizumab + Nivolumab + Chemotherapy	Untreated Metastatic Colorectal Cancer (mCRC)	NCT03414983	II/III	Failed to meet PFS endpoint Ɨ	0.0	N/A	75%	Lenz et al., 2022
Lenvatinib + Pembrolizumab	Treatment Refractory Advanced Endometrial Carcinoma	NCT03517449, NCT04865289, NCT03884101	FDA approved	Active	3.4	6.9	88.9%	Makker et al., 2022, Marth et al., 2021
Lenvatinib + Pembrolizumab	Untreated Advanced/Metastatic Renal Cell Carcinoma	NCT02811861	FDA approved	Active, not recruiting	9.2 (vs. 2nd best arm)	median not reached	82.4%	Motzer et al., 2021
Cabozantinib + Nivolumab	Untreated Advanced/Metastatic Renal Cell Carcinoma	NCT03141177	FDA approved	Active, not recruiting	8.3	not reached	75.3%	Choueiri et al., 2021
Axitinib + Pembrolizumab	Untreated Advanced/Metastatic Renal Cell Carcinoma	NCT02853331	FDA approved	Active, not recruiting	4.3	not reached	67%	Rini et al., 2019 Powles et al, 2020
Axitinib + Avelumab	Untreated Advanced/Metastatic Renal Cell Carcinoma	NCT02684006	FDA approved	Active, not recruiting	5.3 (ITT) 6.8 (PD-L1+)	not reached	71.2%	Motzer et al., 2019 Choueiri et al., 2020

Ɨ Although the PFS endpoint was not met, the PFS rate at 18 months were three times that of standard of care

**Table:** 

Pembrolizumab	PD-1 antibody
Nivolumab	PD-1 antibody
Atezolizumab	PD-L1 antibody
Bevacizumab	VEGFA antibody
Lenvatinib	Tyrosine kinase inhibitor selectively targeting VEGFR1–3, FGFR1–4, PDGFRa/b, c-Kit, and RET
Cabozantinib	Tyrosine kinase inhibitor selectively targeting VEGFR2, RET, MET, and AXL

**Table 2.** Several ongoing/concluded clinical testing of the combination of TGF-β and PD-1/PD-L1 pathway blocakade in solid tumors.

Drug combination	Tumor type	Clinical Trial ID	Phase	Status	Change in PFS (months)	Change in OS (months)	Frequency of grade 3–5 adverse events	References
Vactosertib + Durvalumab	Advanced Non-small Cell Lung Cancer (NSCLC) (PD-L1 Positive)	NCT03732274	I/II	Active, not recruiting	N/A	N/A	15.3% (interim)	Cho et al., 2020
Vactosertib + Durvalumab	Urothelial Carcinoma (Recurrent and Advanced)	NCT04064190	II	Active, not yet recruiting	N/A	N/A	N/A	N/A
Vactosertib + Pembrolizumab	Metastatic colorectal or gastric cancer	NCT03724851	I/II	Active, not recruiting	N/A	N/A	9.1% (3/33)	Kim et al., 2021
Vactosertib + Pembrolizumab	Non-small Cell Lung Cancer (NSCLC) (PD-L1 Positive)	NCT04515979	II	Active, recruiting	N/A	N/A	N/A	N/A
Galunisertib + Durvalumab	Metastatic Pancreatic Cancer	NCT02734160	I	Completed	1.9 (single arm)	5.7 (single arm)	69%	Melisi et al., 2021
Galunisertib + Nivolumab	Advanced Refractory Solid Tumors (NSCLC, Hepatocellular Carcinoma)	NCT02423343	I/II	Completed Ɨ	5.26 (NSCLC) 5.39 (HCC) (single arm)	11.99 (NSCLC) 14.52 (HCC) (single arm)	52% (NSCLC)	https://clinicaltrials.gov/ct2/show/results/NCT02423343
GT90001 + Nivolumab	Advanced Hepatocellular Carcinoma	NCT05178043	II	Active, recruiting	N/A	N/A	N/A	N/A
GT90001 + Nivolumab	Metastatic Hepatocellular Carcinoma	NCT03893695	I/II	Active, not recruiting	N/A	N/A	15% (3/20)	Hsu et al., 2021
M7824 (Bintrafusp Alfa)	HPV Associated Cancers	NCT03427411	II	Active, not recruiting	3.5 (ICB naïve) 1.4 (ICB resistant) (single arm)	19.2 (ICB naïve) 4.4 (ICB resistant) (single arm)	63.3% (ICB naïve) 80.7% (ICB resistant)	https://clinicaltrials.gov/ct2/show/results/NCT03427411
M7824 (Bintrafusp Alfa)	Metastatic Colorectal Cancr or Advanced Solid Tumors	NCT03436563	I/II	Active, not recruiting	1.8 (single arm)	9.1 (single arm)	13.3% (2/15)	Morris et al., 2021
M7824 + HPV Vaccine	HPV Associated Cancers	NCT04432597	I/II	Active, recruiting	N/A	N/A	N/A	Charalampos et al., 2021
M7824	Advanced Untreated Non-small Cell Lung Cancer (NSCLC) (PD-L1 Positive)	NCT03631706	III	Terminated ƗƗ	N/A	N/A	N/A	Ahn et al., 2019
M7824	Checkpoint Inhibitor Naive and Refractory Subjects With Urothelial Carcinoma	NCT04501094	II	Terminated ƗƗƗ	N/A	N/A	N/A	N/A
M7824 + Entinostat and M9241	Advanced Solid Tumors	NCT04708470	II	Active, recruiting	N/A	N/A	N/A	N/A
M7824	Thymoma and Thymic Carcinoma	NCT04417660	II	Active, recruiting	N/A	N/A	N/A	N/A
M7824 + Gemcitabine	Previously Treated Advanced Adenocarcinoma of the Pancreas	NCT03451773	I/II	Terminated ^	N/A	N/A	N/A	N/A
M7824	Operable and Untreated Head and Neck Squamous Cell Carcinoma	NCT04428047	II	Terminated ^^	N/A	N/A	N/A	N/A

Ɨ Due low enrollment, the HCC cohort was terminated early.

ƗƗ Unlikely to meet its PFS primary endpoint when compared to pembrolizumab, trial is discontinued. See https://www.emdgroup.com/en/news/bintrafusp-alfa-037-update-20-01-2021.html

ƗƗƗ Low accrual and safety concern

^ Study was closed after one treatment related death

^^ Sponsor decision following information on cases of hyperprogression and early toxicities with bintrafusp alfa in other studies

**Table:** 

Pembrolizumab	PD-1 antibody
Nivolumab	PD-1 antibody
Durvalumab	PD-L1 antibody
Vactosertib	Tyrosine kinase inhibitor selectively targeting TβRI/ALK5
Galunisertib	Tyrosine kinase inhibitor selectively targeting TβRI/ALK5
M7824/Bintrafusp Alfa	PD-L1 and TGF-β trap antibody
Entinostat	HDAC1/3 deacytelase inhibitor
NHS-IL12	Necrotic tumor region targeting fused with recombinant IL12 cytokine

## Abbreviations:

ICB: Immune checkpoint blockade
IPRES: Innate anti-PD-1 Resistance Signatures
VEGFA: Vascular Endothelial Growth Factor A
TGF-β: Transforming Growth Factor Beta
PD-1: Programmed cell Death protein 1 (PD-1)
PD-L1: Programmed cell Death-Ligand 1 (PD-L1)
CTLA-4: Cytotoxic T-Lymphocyte Antigen 4 (CTLA-4)
